# Ultrasound-Mediated Stimulation of Microbubbles after Acute Myocardial Infarction and Reperfusion Ameliorates Left-Ventricular Remodelling in Mice *via* Improvement of Borderzone Vascularization

**DOI:** 10.1371/journal.pone.0056841

**Published:** 2013-02-20

**Authors:** Jonas Dörner, Rafael Struck, Sebastian Zimmer, Christine Peigney, Georg Daniel Duerr, Oliver Dewald, Se-Chan Kim, Daniela Malan, Thierry Bettinger, Georg Nickenig, Alexander Ghanem

**Affiliations:** 1 Department of Medicine/Cardiology, University of Bonn, Bonn, Germany; 2 Department of Cardiac Surgery, University Clinical Center Bonn, Bonn, Germany; 3 Department of Anaesthesiology and Intensive Care Medicine, University of Bonn, Bonn, Germany; 4 Institute of Physiology I, University of Bonn, Bonn, Germany; 5 Bracco Suisse SA, Geneva Research Center, Plan-les Ouates, Switzerland; Virginia Commonwealth University Medical Center, United States of America

## Abstract

**Aims:**

Post-infarction remodelling (PIR) determines left-ventricular (LV) function and prognosis after myocardial infarction. The aim of this study was to evaluate transthoracic ultrasound-mediated microbubble stimulation (UMS) as a novel gene- and cell-free therapeutic option after acute myocardial infarction and reperfusion (AMI/R) in mice.

**Methods and Results:**

For myocardial delivery of UMS, a novel therapeutic ultrasound-system (TIPS, Philips Medical) and commercially available microbubbles (BR1, Bracco Suisse SA) were utilized in a closed-chest mouse model. UMS was performed as myocardial post-conditioning (PC) on day four after 30 minutes of coronary occlusion and reperfusion. LV-morphology, as well as global and regional function were measured repeatedly with reconstructive 3-dimensional echocardiography applying an additional low-dose dobutamine protocol after two weeks. Scar size was quantified by means of histomorphometry. A total of 41 mice were investigated; 17 received PC with UMS. Mean ejection fraction (EF) prior UMS was similar in both groups 53%±10 (w/o UMS) and 53%±14 (UMS, p = 0.5), reflecting comparable myocardial mass at risk 17%±8 (w/o UMS), 16%±13 (UMS, p = 0.5). Two weeks after AMI/R, mice undergoing UMS demonstrated significantly better global LV-function (EF = 53%±7) as compared to the group without PC (EF = 39%±11, p<0.01). The fraction of akinetic myocardial mass was significantly lower among mice undergoing UMS after AMI/R [27%±10 (w/o UMS), 13%±8 (UMS), p<0.001)]. Our experiments showed a fast onset of transient, UMS-induced upregulation of vascular-endothelial and insulin-like growth factor (VEGF-a, IGF-1), as well as caveolin-3 (Cav-3). The mice undergoing PC with UMS after AMI/R showed a significantly lower scar size. In addition, the microvascular density was significantly higher in the borderzone of UMS-treated animals.

**Conclusion:**

UMS following AMI/R ameliorates PIR in mice *via* up-regulation of VEGF-a, IGF-1 and Cav-3, and consecutive improvement of myocardial borderzone vascularization.

## Introduction

Acute myocardial infarction (AMI) and its sequelae are some of the most common causes of death in the western world, even worldwide. The cicatrization of the infarcted left-ventricular (LV) myocardium leads to morphological and functional changes of the contractile tissue, also referred to as post-infarction remodelling (PIR) [1]. This process is progressive and comprises: a) LV-dilatation, b) deterioration of global and regional LV-function, c) progression of scar size and d) loss of viable myocardial tissue [1], [2]. Each component is associated with increased mortality [3]. Hence, current therapeutic approaches for AMI aim at attenuating PIR [2]. The main objective of our study is to test the functional impact of a novel, non-gene, non-cell based intervention to ameliorate both, morphological and functional changes after AMI and reperfusion in mice.

Clinically, myocardial revascularization is the method of choice in treatment of AMI and the minimization of PIR. Experimental treatment options encompass gene- and cell-based interventions to preserve the contractile performance after AMI. Firstly, myocardial perfusion can be improved by growth factor induced neo-vascularisation (e.g. vascular-endothelial growth factor (VEGF-a) or insulin-like growth factor 1 (IGF-1)) [4]. Secondly, myocardial overexpression of distinct structural proteins (e.g. Caveolin-3, Cav-3) can affect the survival rate of cardiomyocytes after AMI [5]. Previous approaches utilizing gene- and cell-based methods to deliver IGF-1 after myocardial infarction through permanent occlusion of the left coronary artery demonstrated an amelioration of PIR [6]. However, gene- and cell-free methods to stimulate intrinsic overexpression of the mentioned mechanisms are under current investigation [7].

Ultrasound-mediated stimulation of microbubbles (UMS) has been shown to modulate myocardial expression patterns and to improve myocardial transplantation of bone marrow derived cells in rats [7]. However, the additive value of this therapeutic option to reperfusion after AMI in mice has not been elucidated yet.

## Materials and Methods

All experiments have been approved by the animal care committee at the University of Bonn and the local government authorities. Also, they conform to the guidelines of the American Heart Association for the use of animals in research and corresponds to the Guide for the Care and Use of Laboratory Animals published by the US National Institutes of Health (NIH Publication No. 85–23, revised 1985). All animals were housed at a constant room temperature of 24°C and 12 h light–dark cycle and maintained on an *ad libitum* diet.

### Mouse Model of Acute Myocardial Infarction and Reperfusion

#### Coronary instrumentation

**Figure 1:**
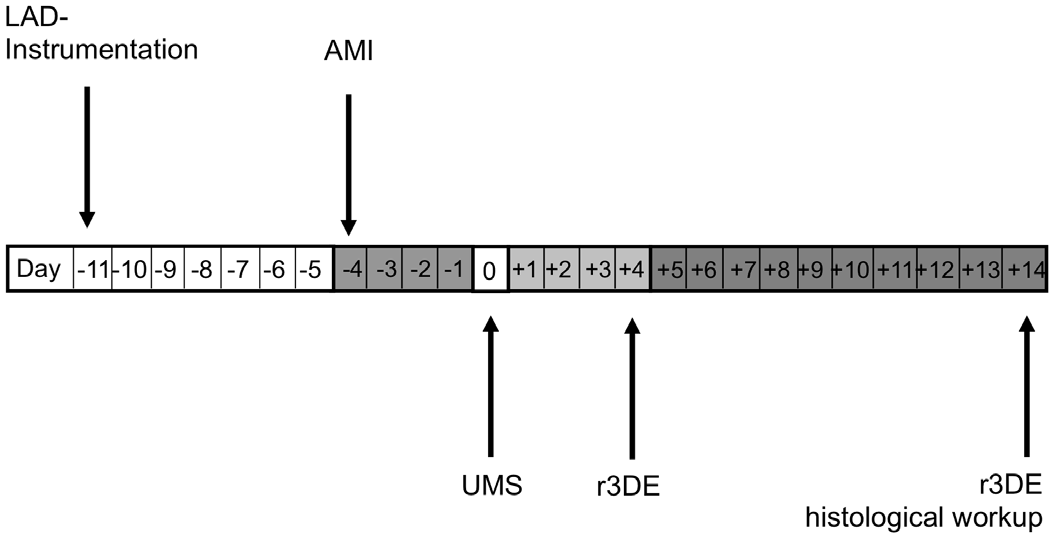
Experimental protocol. The instrumentive surgery of the left anterior descending coronary artery (LAD-Instrumentation) was performed seven days prior to the acute myocardial infarction and reperfusion (AMI/R) to avoid a pro-inflammatory influence of trauma on post-infarction remodelling (PIR). The treatment group received UMS four days after AMI/R. On day +4 and +14, a reconstructive 3-dimensional echocardiography (r3DE) was performed to quantify global and regional left-ventricular function. Additionally, r3DE was carried out with low-dose dobutamine on day +14. Ultimately, hearts were harvested for histological workup.

In order to minimize inflammatory interaction of surgical trauma and AMI, an established closed-chest model of AMI and reperfusion (AMI/R) was utilized (Figure 1) [8]. 56 mice (8–12 weeks old, female, C57BL/6; Charles River, Sulzfeld, Germany) underwent the procedure as recently described [9]. 41 mice were included for functional longitudinal studies and 15 for RNA and protein analysis. Briefly, general anaesthesia was induced with 4% isoflurane (Abbott, Germany) in 1 L per minute O_2_-flow. Mice were intubated with a 22 gauge cannula (Braun, Melsungen, Germany) and connected to a ventilation system (Type Small Animal Ventilator KTR4, Harvard Apparatus GmbH, March-Hugstetten, Germany). Correct intubation and ventilation was confirmed by observing thorax excursions. The animals were placed in a supine position on a feedback heat pad. Their body temperature was measured with a rectal probe and maintained at 36.5°C. The anaesthesia was maintained with 1.2–1.5% isoflurane in 100% O_2_, the animals` heart rates were monitored and kept within physiological ranges to minimize cardio-depressant effects. The respiratory rate was set at 115/min and 10 µL tidal volume per gram body weight. Sufficient analgesia was determined by testing the rear foot reflex.

A left lateral thoracotomy was performed in the forth intercostal-space. The pericardium was opened and gently removed to identify the left anterior descending coronary artery (LAD). The LAD was cautiously under-stitched with an 8–0 prolene suture (Mopylen, Resorba Wundversorgung GmbH & Co. KG, Germany) with a U-shaped tapered needle 1 mm distal of the left auricle. The suture was cut at the needle side and both ends were threaded through 1.0 mm section of a PE 10 tube to form a loose snare around the LAD. To check the correct position of the LAD ligature both ends were transiently tightened. If the position of the ligature was correct paleness of the distal antero-lateral myocardial segments could be observed. Both ends were exteriorized one through the 3^rd^, the other through the 5^th^ intercostal space and stored subcutaneously. The thorax was closed with a prolene suture (6–0, Mopylen, Resorba Wundversorgung GmbH & Co. KG, Germany).

After closing the skin, the mice were weaned from the ventilator and kept in a warm cage. Post-operative care comprised fentanyl 0.1 µg/g bodyweight bid s.c. for analgesia and 7.5 mg/kg bodyweight of the antibiotic enrofloxacine (Baytril™, Bayer Healthcare, Leverkusen, Germany) s.c. for 5 days.

#### Myocardial infarction and reperfusion

For our AMI/R experiments myocardial infarction took place seven days after coronary instrumentation (Figure 1). The Mice were anesthetized with ketamine (65 mg/kg bodyweight), xylazine (13 mg/kg bodyweight) and atropine (0.05 mg/kg bodyweight) i.p. as published previously [10]. The Mice were intubated, ventilated with room air and placed supinely on a feedback heat pad. The skin was re-opened and the exteriorized coronary snare was gently relieved from subcutaneous tissue. Then, tension was carefully applied to achieve controlled closure of the LAD. In addition to the electrocardiogram, an onset of new regional contraction abnormality was monitored using high-resolution echocardiography (Philips HDI-5000, Philips Healthcare). Reperfusion was initiated after 30 minutes by release and gentle removal of the coronary snare. Sham-operated mice underwent the same procedures except for pulling the coronary snare. The skin was closed with a prolene suture (6–0, Mopylen, Resorba Wundversorgung GmbH & Co. KG, Germany).

### Ultrasound-mediated Stimulation of Microbubbles

**Figure 2:**
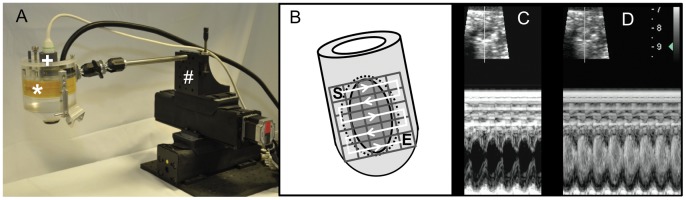
Therapeutic imaging probe system (TIPS) for ultrasound mediated stimulation of microbubbles. (A) Hybrid ultrasound probe system with electromechanically coupled diagnostic (+) and therapeutic probe (*) enabling simultaneous high-resolution imaging for targeted application of UMS. To allow a standardized application to a small, moving target organ, the system is coupled to a computer-programmed stepper motor (#). (B) “En face” heart model to visualize the computer-programmed grid on the anterolateral heart wall. The grid consists of 25 pulses total, administered every 1 mm and 5 pulses per row, respectively starting basal (S) and following the white line to the apex till E (end). UMS was targeted on the anterior left-ventricular wall and the anterior borderzone tissue (area within the dotted circle) after anterolateral ischemia. (C)+(D) Ultrasound B-Mode (upper image) and M-Mode (lower image) short axis view before (C) and after (D) microbubble application in a mouse without myocardial infarction. Hence, this hybrid scanhead allows standardized and targeted myocardial delivery of UMS in mice.

The ultrasound-mediated stimulation of microbubbles (UMS) allows myocardial post-conditioning (PC) by locoregional stimulation of commercially available ultrasound contrast agent with high-intensity focussed ultrasound. For UMS, BR1 (Bracco Suisse SA, Geneva, Switzerland) [11] was continuously infused into a femoral vein which was cannulated with a 27 gauge Viggo™ (Braun, Melsungen, Germany) [7], [12]. UMS was performed with a newly developed, combined ultrasound device (Therapy Imaging Probe System (TIPS); Philips Research North America, Briarcliff Manor, NY, USA) which provides morphological and functional visualisation of target structures with high-resolution b-mode ultrasound imaging and simultaneous and stereotactically coupled application of focussed low-frequency ultrasound (1 MHz, 10000 cycles, 0.3 MPa peak negative pressure, 0.2 Hz) (Figure 2) [13]. A computer-controlled movement ensured the precise and automated UMS application to the LV-myocardium within a stereotactic grid, and was planned using a b-mode short axis view of the left ventricle. The target volume had a total surface of 25 mm^2^ consisting of 5×5 target points with a gap of 1 mm and was placed into the anterior LV-wall aimed at the infarcted area. The ultrasound pulse was delivered with a frequency of 0.2 Hz to allow a sufficient replenishment of the contrast agent. The Control procedure was insonication protocol prior to infusion of microbubbles, as described previously by Miller and co-workers [14].

Additional experiments were conducted to confirm the myocardial delivery of UMS. Evans blue (EB) (Sigma-Aldrich Chemie GmbH, Munich, Germany) is a dye that binds on albumin and therefore stays within the intravasal compartment. Extravasation is an indicator of increased capillary permeability [14]. To evaluate the effect of ultrasound emission power we investigated capillary permeability by means of EB extravasation and transendothelial penetration of fluorescent nanospheres, as published previously [15]. Briefly, we investigated the impact of UMS treatment with different ultrasound force amplitudes on capillary permeability utilising the TIPS-System. Therefore 50 mg/kg EB dye was injected via the femoral vein prior to UMS application [16]. Immediately after UMS, hearts were harvested and rinsed in cardioplegic solution to arrest the heart in end-diastole. Both atria were dissected and the heart was divided into the anterior and the posterior LV-wall. Then both parts were dried for 24 h in 37°C. EB was extracted using 8 µl formamide 99% (Sigma-Aldrich Chemie GmbH, Munich, Germany)/mg tissue as described previously [14], [15]. EB concentration was determined by spectrophotometry of the supernatant at 620 nm against a formamide 99% blank using an absorbance reader (Tecan Safire^2^, Tecan Group Ltd., Switzerland). EB extravasation was calculated as µg EB per mg dry tissue, thus revealing the level of transendothelial extravasation within the targeted myocardial tissue [15].

UMS shows transient biological effects and allows nanoparticles to be delivered transendothelially across sites of UMS-induced transient pore formation [17]. To evaluate efficacy and consistency of delivery to a distinct myocardial target, in this case the LV anterior wall in mice, we used fluorescent nanospheres (Duke Scientific, Palo Alto, CA, Diameter: 30 nm) to visualise the targeted site [17].

Nanospheres were injected simultaneously with microbubbles and UMS protocol was administered with different peak pressures (0.3; 0.5; 1.5 and 3 MPa). Hearts were harvested directly after the administration of UMS and processed for histological analyses. Nanosphere delivery was determined with overlap fluorescent and reflected light microscopy using an Olympus BX 41/Color View II System and Cell-P software (Carl Zeiss MicroImaging GmbH, Germany).

### Reconstructive 3-dimensional Echocardiography (r3DE)

Morphological and functional imaging was performed 4 and 14 days after the application of UMS. All mice were anesthetized with isoflurane (4% for induction, 0.5–0.9% in 1 L per minute O2-flow for maintenance) in 100% oxygen by facemask to avoid cardiodepression and ensure near physiological heart rate. Echocardiography was performed with a commercially available high-resolution ultrasound system (Philips HDI-5000, Philips Healthcare) equipped with a linear-array transducer (CL15-7) operating at 15 MHz and providing frame rates up to 284 Hz. A parasternal long-axis image was used to guide the perpendicular angulation of the transducer for acquisition of the short-axis slices. Then, sequenced 2D, parallel short-axis images of the left ventricle were obtained in 500 µm steps from the aortic root towards the apex by means of a micrometer-screw driven tripod. Ten to fourteen short- axis segments were recorded depending on the overall size of the left ventricle [18], [19]. Parasternal short-axis views were visually divided into six segments. Imaging was considered adequate when the endocardial and epicardial borders could be properly visualized in at least five segments. Cineloops of 50 frames covering minimum two heart cycles were stored digitally and analysed off-line. In addition, we repeated the imaging protocol with an intravenous low-dose dobutamine (10 µg/kg/min) application at day 14 [19], [20].

#### Echocardiographic image analysis

An experienced investigator performed echocardiographic analyses blinded to treatment strategy. End-diastolic measurements were obtained at the peak of the R-wave, whereas end-systolic measurements were obtained at the time of minimum internal chamber dimensions. The acquired sequential 2D short-axis cineloops of the left ventricle were used to measure LV-volumes at end-diastole and end-systole and ejection fraction (EF). Myocardial compartments were differentiated by visual assessment following established echocardiographic criteria: [8] a) akinetic/dyskinetic wall motion (collagenous scar): thinned, echodense wall, no systolic wall thickening, no inotropic response to dobutamine; b) hypokinetic wall motion (peri-infarction borderzone myocardium): normal myocardial thickness, markedly reduced systolic wall thickening during baseline, reduced inotropic response to dobutamine; c) normokinetic wall motion (remote myocardium): normal myocardial thickness, good systolic wall thickening, good inotropic response to dobutamine. Due to its best correlation with the histomorphological scar, wall motion abnormalities are reported as the fraction of “akinetic myocardial mass” [19].

### Reverse Transcriptase Polymerase Chain Reaction

For investigation of expression patterns we varied applied peak pressure (0.3; 0.5; 1.5 MPa) and harvesting time (15 min, 6 h, 30 h) in mice undergoing LV-myocardial UMS. Animals with administration of contrast agent following the insonication protocol served as controls [14]. Also, specific effects of contrast agent only and ultrasound application without contrast agent were investigated as control groups. After hearts were excised, all adnexa were gently removed inclusively the left and right atria, and finally the tissue was recovered in RNAlater™ (Qiagen, Ambion, Inc., Austin, Texas) and stored at 4°C. The tissue was homogenized with a tissue-tearer and mRNA was extracted with the PureLink™ RNA Mini Kit (Invitrogen, Ambion, Inc., Austin, Texas) following the manufactureŕs protocol. cDNA was produced using the High-Capacity cDNA Reverse Transcription kit™ with RNase inhibitor (Applied Biosystems, Foster City, CA, USA) as described by the manufacturer. The PCR was performed as a quantitative measurement with a Taqman™ cycler and a Taqman™ specific real-time PCR kit (TaqMan™ Gene Expression Master Mix (Applied Biosystems, Foster City, CA, USA)). We investigated the time course of expression patterns with three primers: 1. vascular-endothelial growth factor-a (VEGF-a), 2. insulin-like growth factor-1 (IGF-1) and 3. caveolin-3 (Cav-3) (Applied Biosystems, Foster City, CA, USA). The results were calculated as 1/2 ddCt in relation to the housekeeping gene glyceraldehyde 3-phosphate dehydrogenase (GAPDH) (Applied Biosystems, Foster City, CA, USA) after normalization to control mice.

### Enzyme-linked Immunosorbent Assay (ELISA)

To determine the protein expression of IGF-1, the myocardial tissue was homogenized and incubated on ice for 5 min in 1 mL of ELISA buffer containing 20 mM TRIS-HCL, 50 mM sodium chloride, 50 mM sodium fluoride, 10 mM EDTA, 20 mM sodium pyrophosphate, 1 mM Triton X-100 (Sigma-Aldrich, Taufkirchen, Germany) in aqua bidest with protease inhibitors (Complete Mini Tab, Roche, Mannheim, Germany) per 100 mg tissue. The samples were incubated on ice for 10 min and centrifuged for 15 min at 4°C at 14000 rpm. The supernatant was used for measuring myocardial protein levels of IGF-1 (R&D Systems, Minneapolis, USA) using a Tecan Safire^2^ Reader (Tecan Group Ltd., Switzerland). We investigated the protein content [pg/mg myocardial tissue] in: 1. control-groups without any treatment; 2. instrumented mice with AMI/R but without UMS; 3. instrumented mice with AMI/R and with UMS; 4. UMS only.

### Histomorphometric Analyses and Immunohistochemistry

**Figure 3:**
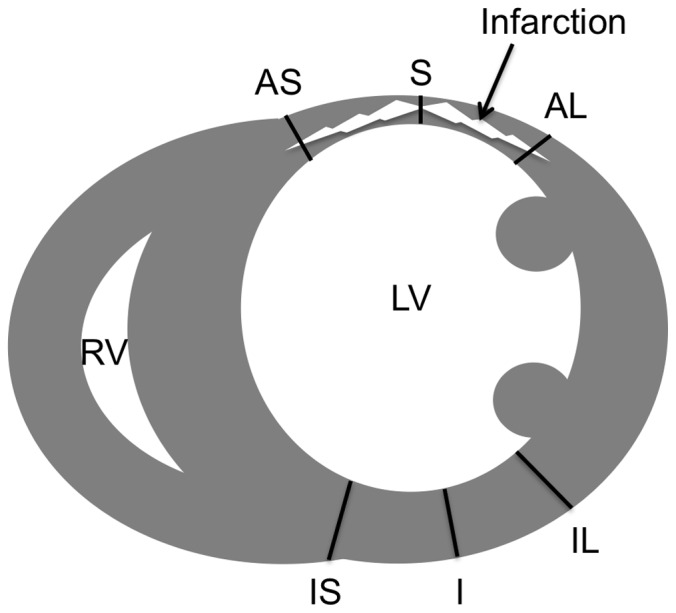
Schematic illustration for determination of hypertrophy. The illustration shows the six locations of wall thickness measurements in a mid-ventricular histological short axis section with scarring of the anterolateral left-ventricular wall. S: scar; AS: anteroseptal borderzone; AL: anterolateral borderzone. Control regions: IS: inferoseptal wall; I: inferior wall; IL: inferolateral wall.

Two weeks after the UMS-application heart samples were prepared for microtome sectioning by rinsing directly in cardioplegic solution after harvesting. Hearts were cannulated via the ascending aorta with a modified Langendorff-system and retrogradely perfused with 4% paraformaldehyde (Merck KGaA, Darmstadt, Germany) at 100 mm Hg pressure for 10 min to avoid the collapse of the left-ventricle [7]. After tissue fixation with 4% paraformaldehyde (Merck KGaA, Darmstadt, Germany) in 0.1 M PBS for 12 h, hearts were incubated in 18% sucrose solution for 6 h [21]. Heart samples were snap-frozen at −80°C in Tissue-Tek™ (Sakura Fintek, Zoeterwoude, Netherlands) and 7 µm slices were prepared with a Kryotom™ (Leica Microsystems GmbH, Wetzlar, Germany). The hearts were cut in transversal direction with 500 µm distance between each stage to be able to measure a total volume by summation of the areas of each transversal section. Slides were stained with picrosirius-red for the demarcation of fibrotic scar and counterstained with fast-green [19]. Sections were fixed and photographed with an Olympus BX 41/Color View II System and Cell-P software (Carl Zeiss MicroImaging GmbH, Germany). Quantitative histomorphometric analyses of scar formation were performed as described previously [22]. Briefly, the ratio of collagenous tissue was analysed using three-dimensional reconstruction with image analysis software (ImageJ; Rasband, W.S., ImageJ, U. S. National Institutes of Health, Bethesda, Maryland, USA). Epicardial and endocardial contours, as well as scar, borderzones and remote regions and their epicardial courses were traced. LV-surfaces, volumes and masses were calculated based on 3D reconstruction of LV-geometry by method of disks [22]. For each section surfaces and masses were calculated by multiplication of areas and circumferential sections with the intersection interval (0.5 mm), respectively. Summarized along the long-axis, those resulted in myocardial volumes and surfaces. Masses were the product of volumes and 1.05 (density of myocardial tissue). For the evaluation of myocardial hypertrophy cross-sectional wall thickness of scar, both adjacent borderzones, and three locations on the posterior wall as a control region were measured (Figure 3). From these measurements the ratio scar/borderzone was calculated by dividing the scar thickness by the mean thickness of the anteroseptal and anterolateral segments.

To evaluate microvascular density CD31 immunohistochemistry was performed. Therefore cryosections were pre-treated with citrate for 15 min in the microwave oven, incubated for 30 min in hydrogen peroxide 3%, and rinsed two times in PBS pH 7.4 for 5 min. Then they have been blocked with the Vectastain rat kit (Vector Laboratories, Burlingame, CA, USA) for 15 min. CD31 staining was performed using the rat anti-mouse CD31 antibody (PECAM-1 monoclonal rat anti-mouse, Dianova, Germany) over night at 4°C with a dilution ratio of 1∶50 in an IgG-block. As a secondary antibody biotin was incubated for 30 min. The antibody was detected with peroxidase using the Vectastain Rat ABC kit (Vector Laboratories, Burlingame, CA, USA) for 30 min and developed with diaminobenzol (DAB) and nickel for 5 min. Microvascular density was assessed by counting the number of CD31-positive vessels in the region of interest. Results were collected as vessels per mm^2^ in the scar, both borderzones, and the posterior wall [23].

### Statistical Analysis

Statistical significance between treatment groups was tested by means of the unpaired *t-test*. Comparison over time, as well as between rest and stress, were tested with the paired Student’s *t-test.* A value of *p*<0.05 was considered significant. Data are indicated as mean± SD.

## Results

A total of 41 C57BL6 mice undergoing AMI/R were randomized into two treatment groups receiving either UMS (n = 17) or control procedure (n = 24). The functional and morphological impacts of UMS on cardiac function after experimental AMI/R were repetitively investigated by means of r3DE on day 4 and 14. Echocardiographic left-ventricular dimensions are displayed in table 1. In both treatment groups, mice revealed a mildly decreased LV-function early after AMI/R prior UMS. At that point of time, LV-EF values were comparable in both treatment groups (53%±10 (w/o UMS), 53%±14 (UMS), p = ns). However, by day 14, untreated mice displayed a significant deterioration of global LV-function (39%±11, p<0.01), as compared to UMS-treated mice (53%±7, p = ns).

**Table 1:** Echocardiographic derived data set.

Day 4	− UMS	+ UMS
LVEDV [µl]	56.95+/−6.08	60.80+/−10.46†
LVESV [µl]	25.22+/−7.47	27.74+/−12.51†
SV [µl]	31.74+/−4.86	31.98+/−5.76†
EF [%]	53.5+/−10.16	52.6+/−14.04†
Heart rate [min^−1^]	501+/−20.44	478+/−22.01†
Cardiac output [ml/min]	15.90+/−2.40	15.83+/−2.85†
WT scar [mm]	0.78+/−0.06	0.86+/−0.05†
WT inferior [mm]	0.94+/−0.05	1.06+/−0.09‡

**Day 14**	**− UMS**	**+ UMS**
LVEDV [µl]	73.43+/−12.39	51.20+/−6.28#
LVESV [µl]	47.64+/−15.37	23.26+/−4.63#
SV [µl]	25.79+/−5.65	27.94+/−2.61†
EF [%]	39.2+/−10.9	53.2+/−7.28#
Heart rate [min^−1^]	480+/−23.10	498+/−41.58†
Cardiac output [ml/min]	12.42+/−2.98	13.91+/−1.64†
WT scar [mm]	0.73+/−0.05	0.86+/−0.05#
WT inferior [mm]	0.94+/−0.05	1.02+/−0.08†
**Day 14 (dobutamine)**	**− UMS**	**+ UMS**
LVEDV [µl]	61.71+/−12.54	50.38+/−5.45†
LVESV [µl]	29.93+/−11.33	17.64+/−3.19†
SV [µl]	31.78+/−6.77	32.74+/−6.76†
EF [%]	53.1+/−12.32	61.8+/−11.17†
Heart rate [min^−1^]	504+/−26.63	550+/−57.17†
Cardiac output [ml/min]	16.0+/−3.41	17.81+/−2.84†
WT scar [mm]	0.75+/−0.08	0.88+/−0.08‡
WT inferior [mm]	0.94+/−0.05	1.08+/−0.04#

Legend:

† p>0.05 compared to −UMS.

‡ p<0.05 compared to −UMS.

# p<0.01 compared to −UMS.

LVEDV: Left-ventricular enddiastolic volume; LVESV: Left-ventricular endsystolic volume; SV: Stroke volume; EF: Ejection fraction; WT: Wall thickness.

**Figure 4:**
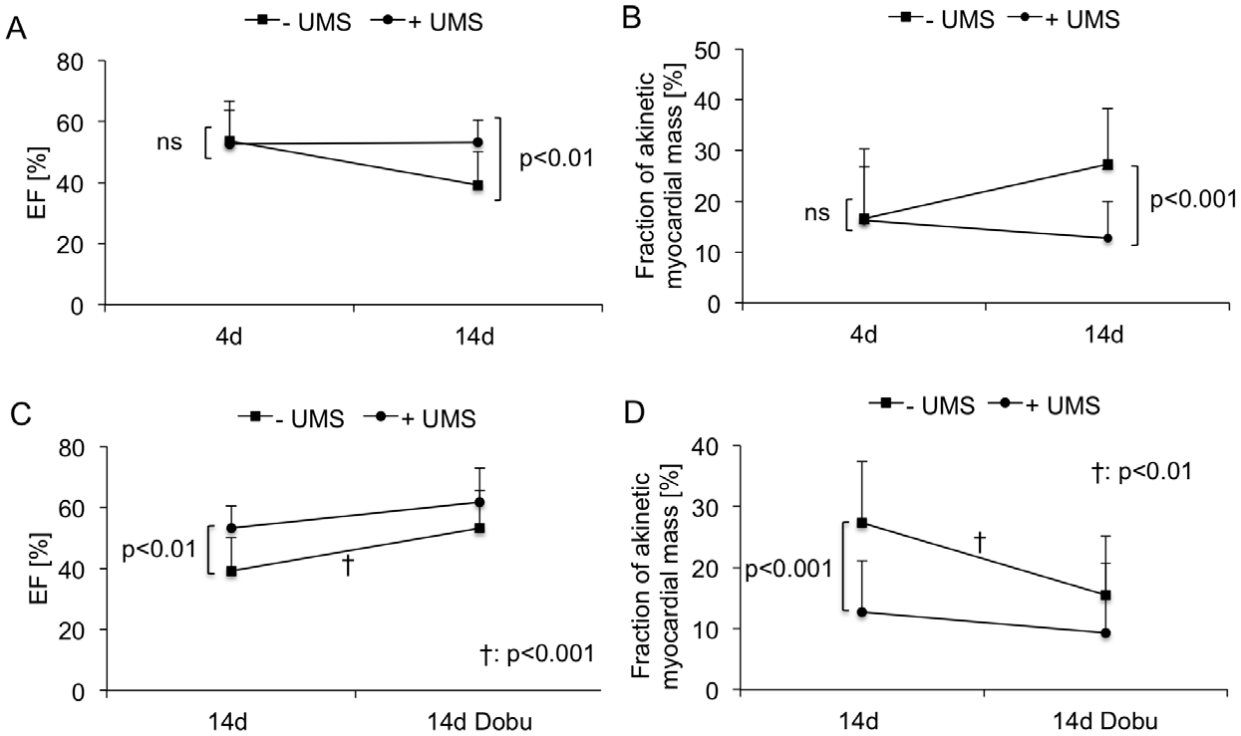
Global and regional left-ventricular function. (A) Mean left-ventricular ejection fraction (LV-EF) was moderately reduced four days after acute myocardial infarction and reperfusion (AMI/R) prior to ultrasound-mediated stimulation of microbubbles (UMS) in both groups. (B) Regional LV-function was quantified by means of reconstructive 3-dimensional echocardiography (r3DE) and is expressed as fraction of akinetic myocardial mass. UMS-treated animals (circles) demonstrated functional improvement two weeks after AMI/R, as compared to controls (squares). (C, D) Global and regional LV-function were obtained prior and during pharmacological stimulation with low-dose dobutamine on day +14. Inotropic response was preserved in both groups and revealed a significant increase in LV-EF and decrease in fraction of akinetic myocardial mass. Both non-invasive measures are parameters indicating preserved myocardial viability after AMI and reperfusion. In all, UMS improved LV-function after AMIR/R without impact on myocardial viability.

In order to estimate regional LV-function, the akinetic mass of LV-myocardium was quantified by means of r3DE. In parallel, on day 4 there were no significant differences between both groups (17%±8 (w/o UMS), 16%±13 (UMS), p = ns). However, by day 14 the akinetic myocardial mass of UMS-treated mice remained stable (13%±8), whereas the control group demonstrated a significant increase (27%±10, p<0.001) (Figure 4 A+B). To further investigate global and regional LV-function, mice were subjected to low-dose dobutamine. During pharmacological stimulation two weeks after AMI/R, no significant differences between both groups were observed (Figure 4 C+D). Left-ventricular ejection fraction as a parameter for global LV-function increased during low-dose dobutamine reflecting inotropic myocardial recruitment. Concordant results were obtained for the quantity of akinetic myocardial mass. During inotropic stimulation, both groups revealed a substantial decrease of akinetic myocardial mass (Figure 4 D). In all, UMS had no additional impact on contractile reserve during pharmacological stimulation.

**Figure 5:**
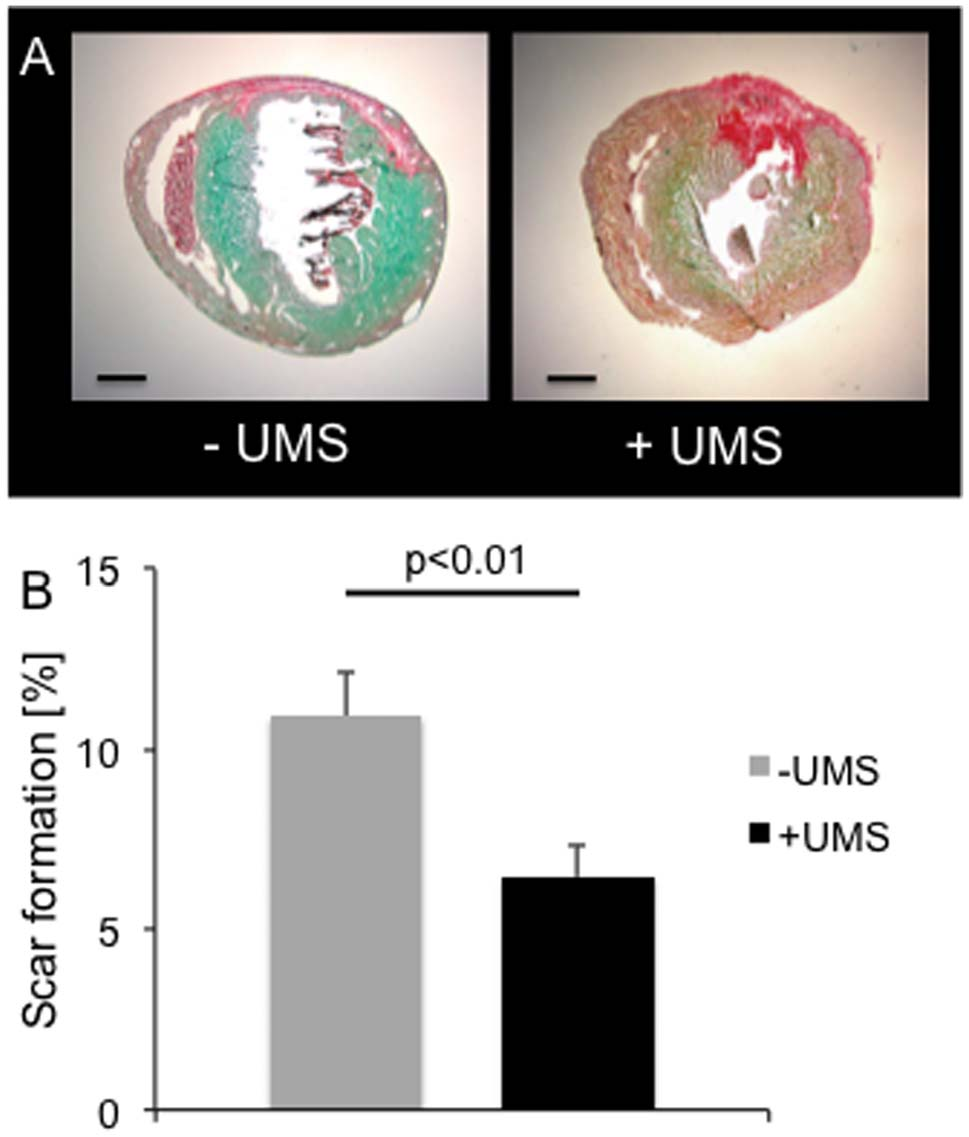
Histological analyses. Myocardial scar formation was determined by means of histomorphometry. (A) Representative images of short axis mouse heart sections stained with picrosirius-red and fast-green without (left) and with (right) UMS-treatment. The collagenous tissue is coloured red whereas myocardium is green. (B) UMS-treated mice demonstrated a significantly lower collagenous scar burden compared to control mice.

Histological analyses of scar size were concordant with functional results. We observed a significant reduction of myocardial scar burden in mice treated with UMS (w/o UMS: 10.9%±5.8; UMS: 6.5%±3.7, (p = 0.006), Figure 5). Data derived from histomorphometric analysis demonstrate higher wall thickness of scar and borderzone tissue in mice treated with UMS. Remote regions had comparable dimensions (see table 2). Our histological data correspond well with the echocardiographic measurements. To our knowledge, this is the first report of amelioration of PIR utilizing an organ-targeted, non-cell- and non-gene-based intervention in mice.

**Table 2:** Evaluation of hypertrophy derived from histomorphometric data.

	− UMS	+ UMS
Myocardial scar [mm]	0.72+/−0.20	0.91+/−0.27#
Anteroseptal left-ventricular WT [mm]	1.19+/−0.26	1.30+/−0.28†
Anterolateral left-ventricular WT [mm]	1.31+/−0.25	1.35+/−0.24†
Inferoseptal left-ventricular WT [mm]	0.83+/−0.21	0.88+/−0.17†
Inferior left-ventricular WT [mm]	0.96+/−0.18	0.94+/−0.30†
Inferolateral left-ventricular WT [mm]	1.00+/−0.20	1.06+/−0.21†
Ratio inferior	1.06+/−0.10	0.95+/−0.19#
Ratio scar/borderzone	0.59+/−0.18*	0.69+/−0.19† *
Heart weight [mg]	82.3+/−12.2	90.7+/−14.8†
Body weight [g]	20.5+/−1.8	21.9+/−2.3†
Normalized heart weight [mg/g]	4.01+/−0.32	4.13+/−0.32†

Legend:

WT: Wall thickness.

† p>0.05 compared to **−** UMS.

# p<0.05 compared to **−** UMS.

* p<0.001 compared to “ratio inferior” as the control region in the same group.

**Figure 6:**
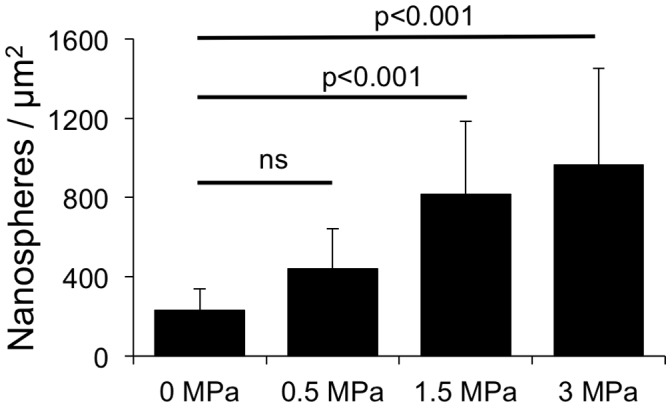
Delivery of myocardial UMS in mice. UMS-mediated myocardial delivery of fluorescent nanospheres was measured and quantified by fluorescence microscopy. Myocardial delivery of fluorescent nanospheres with UMS was feasible in mice and demonstrated a dose-dependent effect.

To investigate potential mechanisms, further experiments were conducted. We characterized the efficacy of myocardial delivery of UMS utilizing EB and nanospheres. UMS effects were more pronounced in the anterior LV-wall as demonstrated by a significantly higher amount of extravasated EB. This well-known effect of UMS was shown to be dose-dependent, revealing lowest extravasation at low emission power (0.3 MPa). In parallel, the transendothelial distribution across microvascular structures showed a dose-dependent increase (Figure 6). These data demonstrate the feasibility to successfully target UMS at small structures such as myocardium of mice.

**Figure 7:**
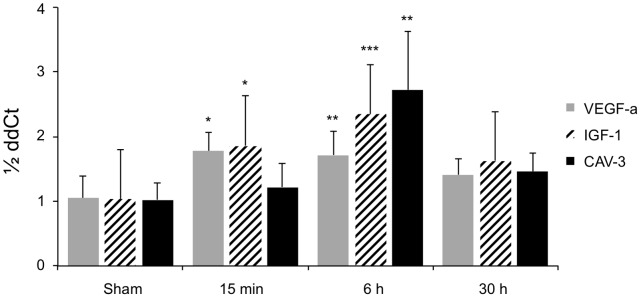
RNA-levels after UMS. UMS increases VEGF-a, IGF-1 and Cav-3 mRNA-levels within 15 min and reaches its peak expression after 6 hours. A prolonged upregulation could not be observed longer than 30 hours after UMS as compared to controls. The displayed p-values refer to the comparison with sham-treated animals.

**Figure 8:**
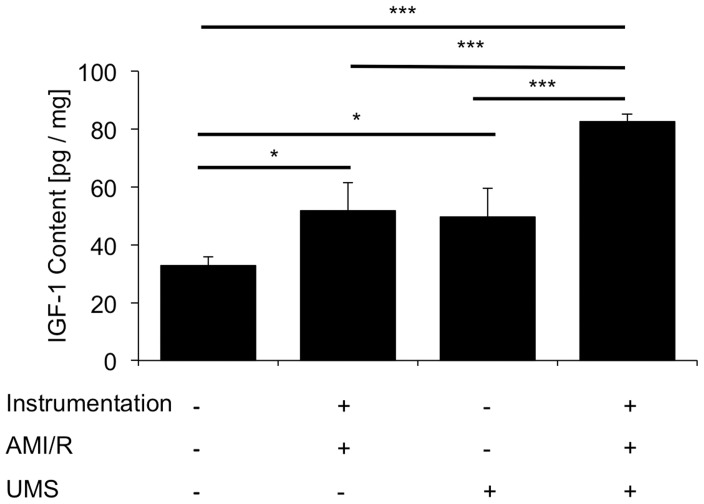
Myocardial protein concentration of IGF-1 after UMS. Insulin-like growth factor 1 (IGF-1) was measured with a quantitative ELISA. UMS application not only increased IGF-1 content in control hearts, but also demonstrated a significant upregulation of IGF-1 on top of acute myocardial infarction and reperfusion (AMI/R).

**Figure 9:**
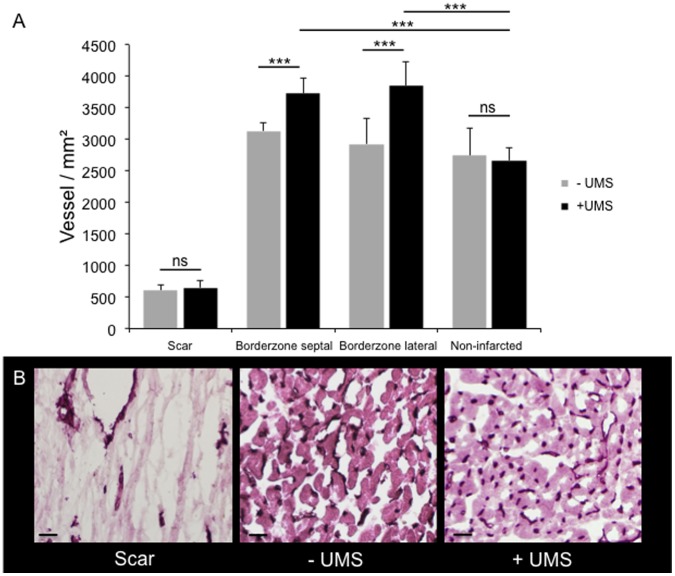
Microvascular density assessed by CD31 staining. (A) Microvascular density was assessed in the myocardial scar, both adjacent borderzones, and non-infarcted regions (posterior left-ventricular wall). No differences between untreated and UMS-treated animals were found with respect to the scar tissue and the non-infarcted regions. However, the myocardial borderzone tissue of UMS-treated mice revealed a significantly increased microvascular density as compared to non-treated animals. (B) Representative CD31 stained histological images of scar, untreated borderzone (−UMS), and UMS-treated borderzone (+UMS) (from left to right). CD31 positive vessels can be identified by their dark colour. Note the higher microvascular density of the UMS-treated mice compared to the non-treated group. In contrast, scar displayed the lowest microvascular density.

To investigate the underlying pathomechanisms, the myocardial expression patterns of three potential mediators were investigated: vascular-endothelial growth factor (VEGF-a), insulin-like growth factor-1 (IGF-1) and caveolin-3 (Cav-3). In general, the mRNA-levels of all three increased transiently within 15 minutes after UMS and reached their maximum after 6 hours (Figure 7). The expression was comparable to the baseline conditions within 30 hours after UMS. Quantitative ELISA confirmed a significant upregulation of myocardial IGF-1 content 18 hours after UMS (Figure 8). Interestingly, UMS was able to increase the IGF-1 protein level by 52% in mice without AMI/R. More important, however, was the significant upregulation by 94% (p<0.001) in the AMI/R group subjected to UMS compared to the AMI/R only group. To further evaluate the potential effect of IGF-1 and VEGF-a on myocardial vascularization, we investigated microvascular density by assessing the number of CD31-positive vessels in post-ischemic and remote myocardial regions of interest. Interestingly, microvascular density normalized to the non-infarcted posterior left-ventricular wall was significantly higher in the borderzone of UMS-treated animals (Figure 9).

Our data demonstrate the ability of UMS to ameliorate post-infarction remodelling *via* modulation of myocardial expression patterns and improvement of borderzone vascularization after AMI/R in mice.

## Discussion

Novel treatment options of PIR focus on early prevention of functional deterioration and scar formation, since myocardial damage caused by AMI have been known to be irreversible. Novel treatment options of AMI with additional benefit to reperfusion include cell- and gene-based strategies [6], [24], [25]. However, both are based on myocardial uptake of foreign material and show limited efficacy of transplantation and transfection, respectively [26]. Hence, a transthoracic therapeutic option modulating intrinsic myocardial expression patterns in PIR is of interest. UMS is a promising non-genetic, non-cell based approach known to influence myocardial expression patterns in rats [7]. However, the exact mechanism of its modulatory effect has not been fully elucidated yet. Previous studies obtained oscillation and microjet formation of insonicated microbubbles and consecutive non-lethal cell alteration and sterile inflammation of the targeted organ [27], [28]. However, functional data on delivery of UMS to even smaller targets after AMI are not available. To our knowledge, this is the first study to demonstrate feasibility and efficacy of UMS in mice following AMI/R.

Since UMS may provide a new treatment option after AMI/R and is able to influence PIR on top of reperfusion, we investigated potential mechanisms of the UMS effect. Firstly, the UMS-mediated overexpression of VEGF-a may help explain functional improvements and amelioration of PIR [29], [30]. VEGF-a plays a substantial role in neovascularization and has been shown to ameliorate PIR in gene- and cell-based studies [4], [31], [32]. Neovascularization improves myocardial function and preserves viability of borderzone myocardium [33]. Secondly, we observed UMS-driven overexpression of myocardial IGF-1. We demonstrated a time-dependent, transient UMS-effect in RNA- and protein analyses after AMI/R. In previous studies, IGF-1 displayed a critical role in PIR, improving myocardial cell survival via PI-3K/AKT activation, which resulted in an improved functional outcome after AMI/R [25], [34]–[36]. Further, it has been reported that combined intra-myocardial application of VEGF-a and IGF-1 is closely related to a better myocardial function and a lower rate of heart failure after acute myocardial infarction in rats [4]. Ultimately, significant overexpression of Cav-3 was induced with UMS. Cav-3 is known to play a critical role in the hypertrophic remodelling of murine myocardium [37]. Therefore UMS-induced overexpression of Cav-3 is another conceivable mechanism of acquired cardioprotection after AMI/R, since UMS results in stable overexpression of Cav-3 and demonstrated the amelioration of PIR [5]. However, additional experiments are necessary to elucidate the value of each contributing factor.

### Limitations

This study is limited by the mechanistic proof of the UMS effect. Only a selection of the known and suspected factors was investigated. However, the experimental setup aimed to demonstrate the proof of principle. Moreover, we aimed at therapeutic targets, which are available for future comparative studies with gene- and cell-based therapeutic approaches. Some challenges will remain crucial for the translation of UMS into larger animal models or even the clinical scenario, two of which are: locoregional delivery of ultrasound energy deep into the body through the skin into a moving organ and coverage of a therapeutically significant volume in a clinically acceptable amount of time. Distinct focused devices are under current development to cover the mentioned requirements and will allow translation into the canine model with comparable penetration depth, target volume, and thoracic impedance as compared to humans. However, from a technical point-of-view, delivery of UMS to a larger organ seems to be simpler than in mice.

### Conclusion

UMS allows a cell- and gene-free amelioration of PIR on top of reperfusion *via* the up-regulation of VEGF-1, IGF-1 and Cav-3 and consecutive improvement of myocardial borderzone vascularization. However, further studies need to elucidate the pivotal mechanism.
